# The long-term straw return resulted in significant differences in soil microbial community composition and community assembly processes between wheat and rice

**DOI:** 10.3389/fmicb.2025.1533839

**Published:** 2025-02-27

**Authors:** Siyuan Cui, Shangqi Xu, Guangqiao Cao, Xinkai Zhu

**Affiliations:** ^1^Nanjing Institute of Agricultural Mechanization, Ministry of Agriculture and Rural Affairs, Nanjing, China; ^2^Collaborative Innovation Center of Recovery and Reconstruction of Degraded Ecosystem in Wanjiang Basin Co-founded by Anhui Province and Ministry of Education, School of Ecology and Environment, Anhui Normal University, Wuhu, China; ^3^Jiangsu Key Laboratory of Crop Genetics and Physiology, Yangzhou University, Yangzhou, China

**Keywords:** straw return, rice-wheat rotation, agricultural ecosystem, soil pH, soil microbial communities, environmental factors

## Abstract

**Introduction:**

Straw return is widely promoted as an environmentally sustainable practice to enhance soil health and agricultural productivity. However, the impact of varying straw return durations on soil microbial community composition and development remains insufficiently understood within a rice-wheat cropping system.

**Methods:**

In this study, soil samples were collected during the wheat and rice harvesting periods following seven straw return durations: no straw return (NR) or 1, 3, 5, 7, 9, 11 years of straw return (SR1, 3, 5, 7, 9, 11), and microbial sequencing was performed.

**Results:**

The results revealed a biphasic pattern in alpha diversity (Chao1 and Shannon) of soil microbial communities with increasing straw return duration, characterized by an initial increase followed by a subsequent decrease. Specifically, SR9 in the rice group exhibited the highest Chao1 and Shannon values, while SR3 in the wheat group showed the highest values. PCoA indicated significant shifts in microbial communities due to straw return, particularly in the wheat group compared to NR. Straw return obvious changed six bacterial phyla (Verrucomicrobiota, Proteobacteria, Desulfobacterota, MBNT15, Actinobacteriota, and Gemmatimonadota) during the rice and wheat harvesting periods, especially Proteobacteria. Correlation analysis between environmental factors and bacterial communities demonstrated a significant impact on these factors, particularly pH and total organic carbon (TOC) (*p* < 0.05), on the soil bacterial community during rice harvest, indicating the microbial enrichment after straw return may be related to the accumulation of TOC. Furthermore, the bacterial community network in the rice harvesting period was found to be more complex, with lower network stability compared to the wheat harvesting period. This complexity is closely associated with TOC accumulation in rice fields. Deterministic processes, including homogeneous and heterogeneous selection, were found to play a crucial role in shaping the soil bacterial communities in both rice and wheat systems. Environmental factors significantly influenced microbial community assembly during straw return and recycling.

**Discussion:**

Our study enhances understanding of the impact of straw return on the diversity and assembly of soil microbial communities in the rice-wheat cropping system, which provide valuable insights for studying the mechanisms by which managing microbial communities after straw return can promote soil fertility restoration.

## 1 Introduction

Straw return is a widely adopted environmentally friendly strategy aimed at safeguarding food supply and improving soil quality (Zhang et al., 2021). Incorporating straw into the soil benefits rice-wheat cropping systems by reducing crack formation, improving soil aggregation, sequestering soil organic carbon (SOC), and mitigating the adverse effects of chemical fertilizers (Zhang et al., 2020; Liu J. et al., 2023; Zhang et al., 2024). Various straw management approaches influence soil fertility and crop yields (Chen L. et al., 2022), with straw return increasingly recommended due to its positive effects on yield and soil quality (Huddell et al., 2020; Liu J. et al., 2020; Yang et al., 2021). Soil properties, microbial communities, enzyme activity, and crop growth are significantly affected by the duration of straw incorporation, especially in multiple cropping systems (Zhu X. et al., 2023).

Several studies have explored the impact of straw return on soil microbial communities and enzyme activities, demonstrating that straw return significantly influences both bacterial and fungal communities. For instance, a 5 years study in a rape-rice rotation system showed that straw return enhances soil structure, carbon and nitrogen levels, and bacterial diversity (Yuan et al., 2023). Short-term studies have reported an increase in bacterial and fungal abundance following rice straw return, with fungi becoming more dominant. Evaluations of varying amounts of rice straw return reveal diverse effects on soil microbial communities, indicating that microbial diversity and composition are influenced by different levels of straw addition (Wang et al., 2021). Long-term straw return combined with fertilization has been found to affect soil properties, microbial communities, enzyme activities, and crop growth under dual cropping systems (Yang L. et al., 2022). Meanwhile, straw decomposition is a intricate process primarily facilitated by specialized soil microorganisms with distinct functions. A diverse range of microbial communities is crucial for the decomposition of crop residues. During the early stages of decomposition, bacteria are particularly effective at breaking down labile compounds and are the dominant agents in the degradation of straw. In contrast, fungi are primarily responsible for decomposing more recalcitrant materials during the later stages of decomposition. The energy and carbon derived from the incorporation of crop residues into the soil are distributed across various trophic levels, thereby enhancing soil health and micro-ecological environment (Yang L. et al., 2022).

Farmland use directly impacts soil microorganism communities. The flood and drainage cycles typical of rice production create alternating reduced and oxidized environments, which support the growth of diverse microbes. Consequently, significant differences in microbial communities exist between upland regions and paddy fields (Sun et al., 2018; Zhu Y. et al., 2023; Coleine et al., 2024). For example, in paddy soil, the abundance of bacteria is significantly greater than in upland soil, especially when it comes to nitrogen-fixing cyanobacteria. However, in comparison to upland soil, the abundance of fungi and actinomycetes is relatively lower in paddie soil (Xia et al., 2020). Therefore, the active microbial community is capable of effectively degrading the rice straw that has been returned into the field. This process, in turn, significantly enhances soil fertility.

Microbial communities are generally influenced by both deterministic and stochastic processes, which are shaped by various abiotic and biotic factors (Davison et al., 2024; Li Y.-Z. et al., 2024). Straw return has been shown to enhance the dominance of stochastic processes in structuring soil fungal communities (Shi et al., 2020). In this context, the practice of straw return does not merely alter the physical and chemical properties of the soil. Instead, it goes a step further and exerts an influence on the health and productivity of soil ecosystems by shaping the composition and functionality of microbial communities.

Previous studies underscore the significant role of straw return in governing soil microbial communities, enhancing soil fertility, and improving crop yields. These findings highlight the importance of sustainable agricultural practices such as straw return for maintaining soil health and productivity. However, the effects of different straw return durations on soil microbial community composition and community assembly remain insufficiently explored in the rice-wheat cropping system, which is a paddy-upland rotation system. This study investigates the impacts of straw return duration on the diversity and assembly of soil microbial communities in a rice-wheat cropping system. An 11 years field experiment was conducted in a rice-wheat cropping system with straw return. The composition and abundance of soil microorganisms were analyzed using 16S rRNA amplicon sequencing. We examined various soil physicochemical properties (e.g., total organic carbon (TOC), total nitrogen (TN), available phosphorus (AP), rapidly available potassium (AK)) and microbial characteristics (alpha diversity and beta diversity) to understand the associations between environmental factors and microbial communities. Additionally, we analyzed the community assembly mechanisms and co-occurrence patterns of soil microbes, provides effective insights on how to optimize straw return management strategies to maximize its ecological benefits.

## 2 Materials and methods

### 2.1 Experimental site

**FIGURE 1:**
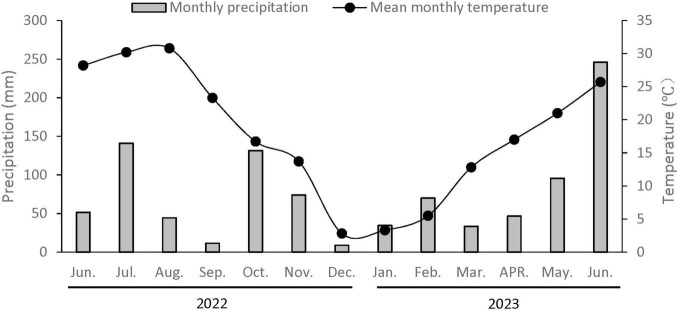
Average monthly air temperature and monthly precipitation throughout the research period at the Yangzhou station (Jiangsu, China).

The rice-wheat rotation field for straw return trials was established in Yangzhou, China, located at coordinates 32°23′ N and 119°25′ E. Straw incorporation began from 2010 and continued for more than 10 years. Weather data for the experimental duration are provided in Figure 1. The soil was classified as a Stagnic Anthrosol (Gong and Chen, 2007), with a pre-experiment bulk density of 1.45 g cm^–^ł, and contained 15.73 g kg^–1^ TOC, 1.24 g kg^–1^ TN, 16.32 mg kg^–1^ AP, and 146.12 mg kg^–1^ AK in the top 20 cm layer. This area is one of China’s main rice-wheat double-cropping zones.

### 2.2 Experimental design

A randomized block design with seven treatments was implemented, including no straw return (NR) and annual straw return for one (SR1), three (SR3), five (SR5), seven (SR7), nine (SR9), and eleven (SR11) consecutive years. Every treatment had four replicates in 4 m × 3 m plots.

Following rice harvests, 9,000 kg hm^–2^ of chopped rice straw (cut into 10 cm pieces) was spread across each plot and tilled into the top 15 cm of soil. Wheat straw was removed after each wheat harvest.

Nitrogen was applied at 240 kg hm^–2^ per season in four stages: as a base, during tillering, jointing, and heading at a ratio of 5:1:2:2. Phosphorus (P2O5) and potassium (K2O) were applied at 90 and 150 kg hm^–2^, respectively, divided equally between the base and jointing stages. Rice received similar treatment, with an additional 150 kg hm^–2^ of urea applied during the tillering and booting stages.

### 2.3 Sample collection and analytical methods

Soil samples were collected using a five-point sampling method after the wheat harvest in October 2022 and again in June 2023. Four replicates were harvested per treatment from a depth of 0–20 cm. Each soil sample was divided into two portions: one was air-dried naturally, sieved through a 0.25 mm mesh to remove gravel and crop residues for environmental factor analysis, while the other was stored at −80°C for future soil DNA extraction.

Soil nutrient parameters such as soil pH, dry-wet ratio, TOC, TN, AP, AK, ammonia nitrogen, nitrate nitrogen, and microbial carbon contents were measured according to the methods outlined by Bao (2000).

Microbial DNA was isolated from soil samples using the E.Z.N.A.^®^ Soil DNA Kit (Omega Bio-tek, Norcross, GA, United States) following the manufacturer’s recommendations. The bacterial 16S ribosomal RNA gene’s V4–V5 regions were amplified via PCR using the following conditions: 2 min at 95°C, followed by 25 cycles of 30 s at 95°C, 30 s at 55°C, and 30 s at 72°C, concluding with a final extension at 72°C for 5 min. The primers used were 341F (5′-barcode-CCTAYGGGRBGCASCAG-3′) and 806R (5′-GGACTACNNGGGTATCTAAT-3′), with the barcode being an eight-base sequence uniquely linked to each sample. PCR reactions were performed in triplicate using 20 μL mixtures that included 4 μL of 5 × FastPfu Buffer, 2 μL of 2.5 mM dNTPs, 0.8 μL of each primer (5 μM), 0.4 μL of FastPfu Polymerase, and 10 ng of template DNA. Amplicons were extracted from 2% agarose gels and purified with the AxyPrep DNA Gel Extraction Kit (Axygen Biosciences, Union City, CA, United States) according to the manufacturer’s instructions.

Purified PCR products were quantified using a Qubit^®^ 3.0 (Life Invitrogen), and equal amounts of every 24 amplicons with different barcodes were combined. The pooled DNA product was utilized to construct an Illumina Pair-End library, following the genomic DNA library preparation procedure outlined by Illumina. The amplicon library was sequenced using paired-end (2 × 300 bp) on an NGS platform at Shanghai BIOZERON Biotech. Co., Ltd., following standard protocols.

### 2.4 Bioinformatics analysis

Raw fastq files were demultiplexed using Trimmomatic (Sun et al., 2024)^1^ and in-house Perl scripts based on the barcode sequence information for each sample Reads that could not be assembled were discarded. Sequences that passed quality control were dereplicated and analyzed using the DADA2 algorithm (recommended by QIIME 2) (Callahan et al., 2016). After merging paired reads and filtering out chimeras, the phylogenetic affiliation of each 16S rRNA gene sequence (ASVs) was assessed using the uclust algorithm (Edgar, 2010)^2^ with the 16S rRNA database from SILVA^3^, applying a confidence threshold of 80% (Quast et al., 2012). The Chao 1 and Shannon indices were used to estimate microbial alpha diversity, whereas microbial beta diversity was determined through Bray-Curtis principal coordinate analysis (PCoA) and analysis of similarities (ANOSIM) using the “vegan package” in R (v.4.0.3) (Xiong et al., 2020). Redundancy analysis (RDA) (Qiu et al., 2021) was conducted to assess the relationships between the bacterial communities and environmental variables. To explore the correlation between changes in environmental factors and differences in bacterial communities, linear regression was employed.

### 2.5 The mechanism of microbial community construction and network analysis

Variation in the phylogenetic or taxonomic diversity of microbial communities was assessed using the weighted beta nearest taxon index (betaNTI) (Stegen et al., 2013) and Bray–Curtis-based Raup–Crick matrix (RCbray) (Stegen et al., 2012). A betaNTI value of less than -2 (indicating homogeneous selection) or greater than +2 (indicating variable selection) indicated the predominance of deterministic processes with a lower or higher phylogenetic turnover than expected. A | betaNTI| value of less than 2, combined with | RC-Bray| values greater than 0.95 and less than -0.95, reflects the relative contributions of dispersal limitation and homogenizing dispersal, respectively. Additionally, a | betaNTI| value of less than 2 and | RC-Bray| values below 0.95 signify the relative contribution of the undominated fraction.

Co-occurrence (Chen C. et al., 2022) networks were developed using the WGCNA package, based on Spearman’s correlation (Siska and Kechris, 2017) matrices derived from 16S rRNA sequencing data, where Spearman’s correlation coefficient was greater than 0.8 and the *p*-value was less than 0.05. Only ASVs with relative abundance > 0.01% and detection rate > 60% were utilized in the analyses to limit false positives. In networks, ASVs were represented as nodes, while correlations between ASVs were edges. Zi-pi (Jiang et al., 2015) analysis was performed using the “Hmisc” package in R to identify keystone taxa with threshold Zi < 2.5 and Pi > 0.62.

## 3 Results

### 3.1 Sample sequencing and alpha diversity

**FIGURE 2:**
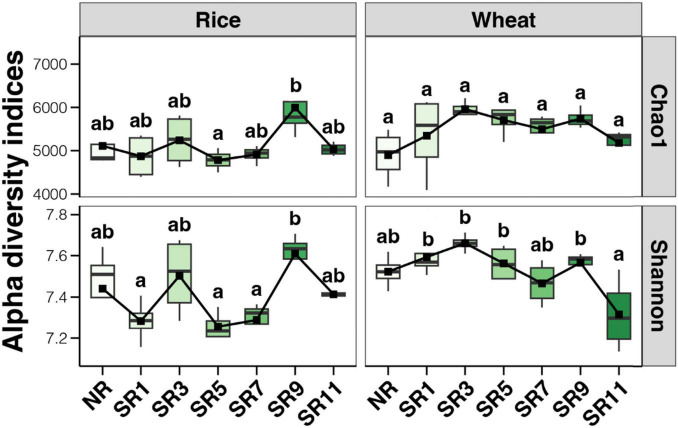
The microbial alpha diversity under different straw return durations of rice and wheat.

A total of 56 soil samples were collected from two crops (rice and wheat) over 2 years (2022 and 2023). To analyze the prokaryotic community, 16S rRNA gene amplicon sequencing was performed, yielding 1,043,313 and 1,006,277 high-quality sequencing reads during the rice and wheat harvest periods, respectively. A total of 13,073 and 13,163 ASVs were isolated by clustering, respectively. Overall, microbial alpha diversity increased to a certain extent following the incorporation of straw into the field, compared to the control group. The alpha diversity of the soil bacterial community fluctuated across different straw return duration treatments during the rice harvest period, being lowest in SR5 and highest in SR9. During the wheat harvest period, the alpha diversity of the soil bacterial community first increased, then stabilized, and finally decreased with the increase in straw return duration. The alpha diversity peaked in SR3, remained relatively stable until SR9, and decreased significantly in SR11 (Figure 2). Straw return and multiple years of incorporation significantly improved soil carbon sequestration, inducing adaptive shifts in the microbial community, including enhanced capacity for organic carbon metabolism, as previously verified (Xu et al., 2024).

### 3.2 Changes in soil bacterial community structure

Principal coordinate analysis (PCoA) based on Bray-Curtis distance was utilized to assess changes in the soil bacterial community structure during rice and wheat harvest stages, as well as to examine the impacts of straw return and different durations of incorporation on microbial community structure in a rice-wheat rotation system.

**FIGURE 3:**
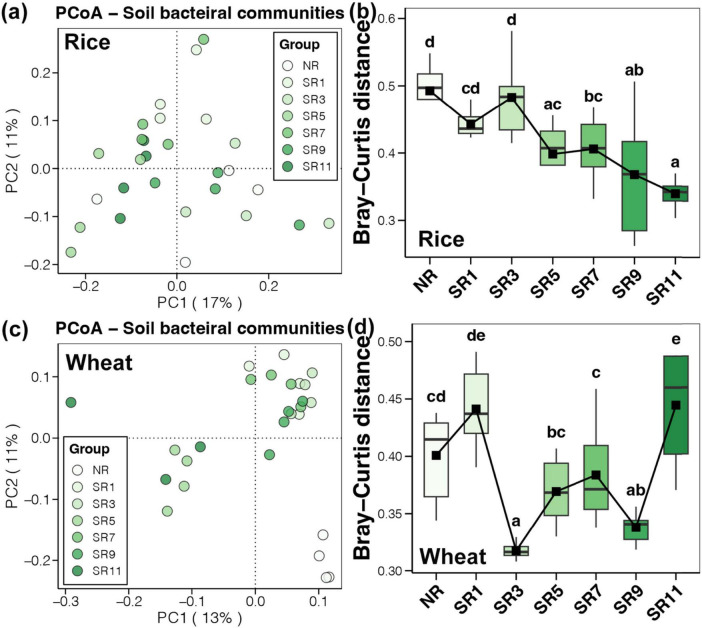
The microbial beta diversity across different straw return durations of rice and wheat. Principal coordinate analysis (PCoA) for soil bacterial communities within rice **(A)** and wheat **(C)**. Microbial community similarity analysis throughout samples, demonstrating the microbial beta diversity in rice **(B)** and wheat soil bacterial communities **(D)**.

Principal coordinate analysis demonstrated no obvious clustering of bacterial community structure in the soil at different straw return durations during the rice harvesting period (Figure 3A). However, comparing the Bray-Curtis distance showed that with increased straw return duration, the variability of soil bacterial community in the same group was reduced (Figure 3B). PCoA indicated that the bacterial community structure in the soil had apparent clustering at different treatments during the wheat harvesting period. The NR treatment was grouped separately, and the SR1–SR11 treatments were grouped into two categories based on various treatments (Figure 3C). Bray-Curtis distance comparisons indicated that the soil bacterial community structure difference was opposite to its alpha diversity at the wheat harvesting stage. At SR3, the difference in soil bacterial community structure was the lowest and remained relatively stable. At SR11, the degree of difference increased significantly and peaked (Figure 3D). The above results suggested that the microbial community composition following straw incorporation significantly differed from the control group, particularly in the wheat group. This can be attributed to the adaptive evolution of the microbial community after straw incorporation. We observed a significant and pronounced alteration in the microbial community during the 3 years straw incorporation, closely related to the level of straw maturity. Moreover, different degrees of straw residue maturation could impact the composition of the microbial community. For example, a study on corn straw composting (Wei et al., 2018), demonstrated that the relative abundance of microorganisms decreased significantly over time, with dominant flora changing during the maturation process.

### 3.3 Soil bacterial community composition

**FIGURE 4:**
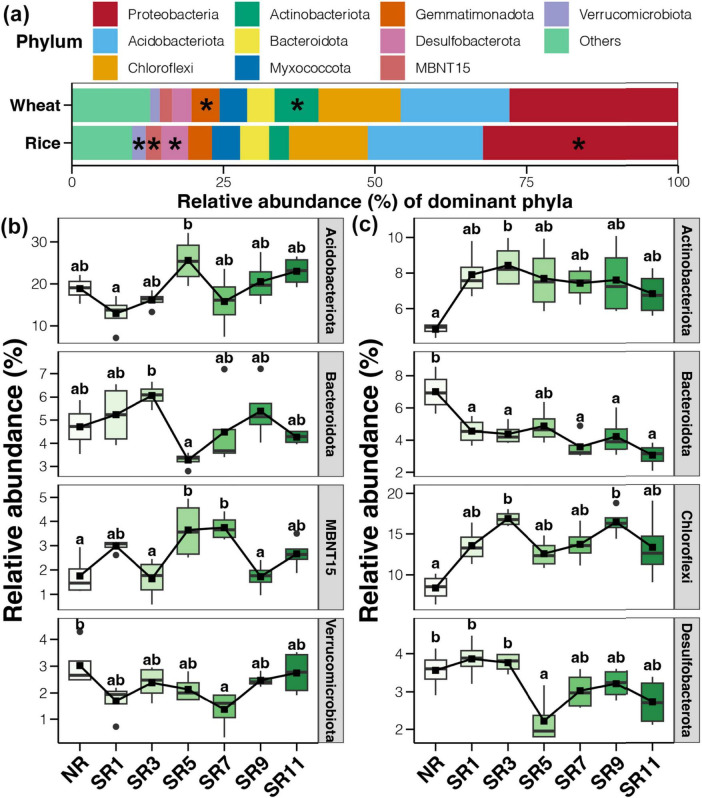
The microbial community structure across different straw return durations of rice and wheat. **(A)** The microbial community makeup at the phylum level. The boxplots of phylum level differences according to Tukey’s HSD test in rice **(B)** and wheat **(C)** soil bacterial communities. The symbol * represents statistical significance.

The enrichment and alteration of dominant bacteria under straw incorporation and incorporation durations were analyzed (Figure 4). The abundance of Proteobacteria, Desulfobacterota, MBNT15, and Verrucomicrobiota in the rice harvesting stage was significantly higher than in the wheat harvesting stage (Figure 4A). However, Actinobacteriota and Gemmatimonadota were found in higher abundance in the soil during wheat harvest. The resistance of actinomycetes and Acidobacteria to drought and high-temperature environments may contribute to this. Additionally, Gemmatimonadota can effectively respond to drought stress in wheat growth and utilize soil humus. Tukey’s HSD test was used to characterize the bacteria affected by straw return duration during rice and wheat harvest, respectively (Figures 4B, C). The results demonstrated that six bacterial phyla (Verrucomicrobiota, Proteobacteria, Desulfobacterota, MBNT15, Actinobacteriota, and Gemmatimonadota) were significantly affected by straw return during the rice and wheat harvesting period. At the rice harvesting stage, Acidobacteriota and MBNT15 first increased and then decreased, and their abundance peaked in SR5. In contrast, Bacteroidota reached its lowest abundance at SR5, whereas Verrucomicrobiota peaked in the sample without straw return and had the lowest abundance at SR7. At the wheat harvest stage, the abundance of Actinobacteriota and Chloroflexi in straw return treatments was significantly higher than in NR, whereas Bacteriodiota was the opposite. Additionally, the abundance of Desulfobacterota decreased significantly in SR5.

The results suggested that the function of the microbial community is key to straw incorporation. Chloroflexi (Peng Z. et al., 2024), Gemmatimonadota (Kong et al., 2022; Peng Z. et al., 2024), Bacteroidota (Wang et al., 2024), Verrucomicrobiota (Xu et al., 2024), Desulfobacterota (Zhou et al., 2024), and other bacteria with substantial changes can utilize carbon and promote the formation of humus from returned straw.

### 3.4 Association of environmental factors with soil bacterial communities

**FIGURE 5:**
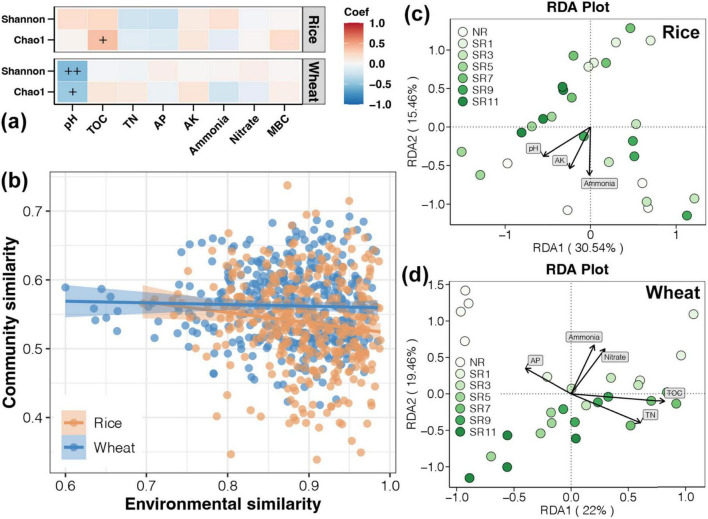
Relationship between environmental factors and soil bacterial communities. **(A)** The Pearson correlation between diverse environmental factors and the alpha diversity index. **(B)** The linear regression analysis of differences in environmental factors and bacterial communities. The redundancy analysis (RDA) of bacterial communities throughout the rice **(C)** and wheat **(D)** harvesting period.

The microbial community composition may undergo significant alterations as the duration of straw incorporation increases, leading to a shift in the dominant environmental factors influencing this community. We used Pearson correlation to determine the relationship between various environmental factors and the alpha diversity index (including Shannon and Chao1) of soil bacterial communities (Figure 5A). The results showed that TOC and Chao1 were significantly positively correlated in the soil at rice harvesting stage, while during the wheat harvest stage, there was a significant negative correlation between pH and alpha diversity. These observations may be attributed to accumulated organic carbon during straw incorporation into the soil and wheat’s inherent resilience to drought and high-temperature conditions. These results indicated that the enrichment of microorganisms in rice straw was quite evident, which led to an increase in the soil TOC content, thereby effectively enhancing soil fertility. Nevertheless, the resistance mechanisms of wheat to drought and high-temperature conditions might result in a suboptimal outcome when it comes to straw return practices. As such, further experimental validation is required to fully understand and address this situation.

From linear regression analysis between differences in environmental factors and bacterial communities, a significant correlation was found between environmental factors and bacterial community only in the rice harvesting period, which was not significant in the wheat harvesting period (Figure 5B). This indicated that environmental factors had a stronger effect on soil bacterial community during the rice harvesting period. The progressive accumulation of organic carbon may exert a more pronounced influence on the structuring of microbial communities as the duration of straw return increases.

RDA was performed to assess the correlation between environmental variables and microbial community. As presented in Figures 5C, D, the bacterial communities were separated into groups corresponding to each section. pH, AK, and Ammonia were the primary forces causing distinct structures of the bacterial communities in rice harvest soil. During the wheat harvest, the AP, TN, TOC, ammonia, and nitrate were the main forces that caused the distinct structures of the bacterial communities. The reintroduction of straw into the field leads to microbial enrichment, specifically associated with carbon metabolism and enhanced resistance to drought and high temperatures, influencing alterations in soil physical and chemical factors.

### 3.5 The coexistence pattern and assembly processes of the bacterial communities

**FIGURE 6:**
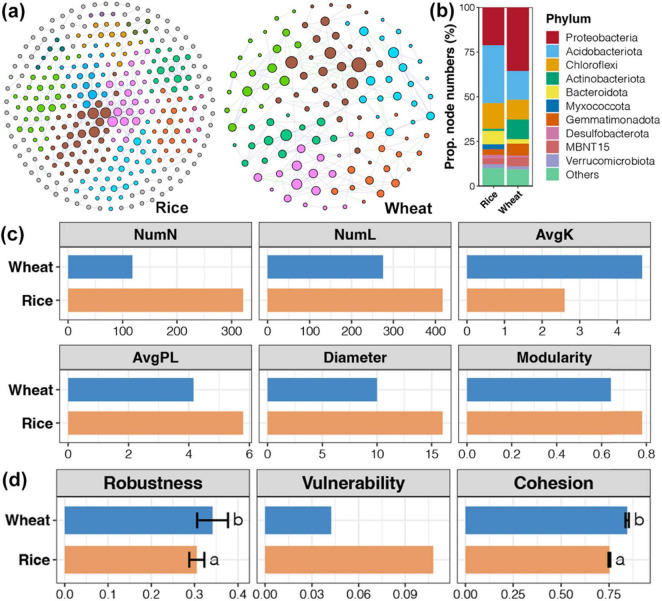
The co-occurrence network of soil bacterial communities throughout the rice and wheat harvesting period. **(A)** A co-occurrence network segregated into phyla differentiated by colors. **(B)** The proportion of OTUs belonging to different bacterial phyla in networks for bacterial communities in rice and wheat soil bacterial communities. **(C)** Diversity in the topological parameters of networks for bacterial communities between rice and wheat soil bacterial communities. **(D)** Variances in stability indices of networks for bacterial communities between rice and wheat soil bacterial communities. NumN, number of nodes; NumL, number of links; AvgK, average degree.

Network analysis was used to construct a soil bacterial community co-occurrence network at the rice harvesting stage and wheat harvesting stage, respectively. The soil bacterial community network at the rice harvesting stage is more complex (Figure 6). In the soil bacterial community co-occurrence network of the rice harvesting period, Acidobacteriota had the highest connection number, while at the wheat harvesting period, Proteobacteria had the highest. Bacteroidota occupied a higher proportion in the network at the rice harvesting stage, while Actinobacteriota occupied a higher proportion at the wheat harvesting stage. By comparing the topological parameters of the network, the rice harvest network had higher nodes and variable numbers, as well as average path length, diameter, and modularity index, which indicated that the rice harvest network was more complex. By contrasting the stability of the network with three parameters, the stability of the network in the rice harvesting period was lower than that in the wheat harvesting period. In the rice system, the microorganisms are more susceptible to straw return and return duration. The growth habits of rice are closely tied to water flooding and the resulting anaerobic environment, inducing rapid microbial flow and significant changes in community composition (Wei et al., 2022). Moreover, flooded conditions during rice cultivation facilitate humus maturation and enhance soil microbial biomass carbon (MBC) and biomass nitrogen (MBN) storage.

**FIGURE 7:**
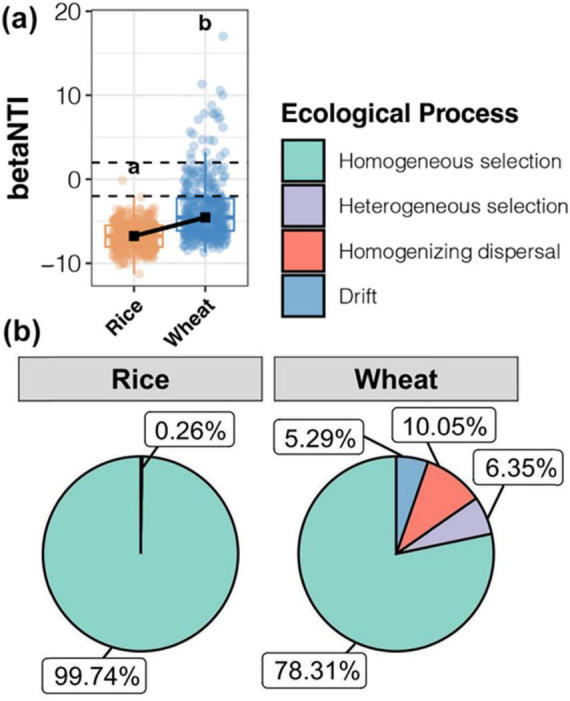
Null model analysis revealed the assembly mechanism of the bacterial communities throughout the rice and wheat harvest soil samples. **(A)** The betaNTI value of the bacterial community. **(B)** Proportion of bacterial assembly types.

Mechanisms of soil microbial community establishment in the rice-wheat system were examined across different durations of straw incorporation. The null model was applied to determine the contributions of ecological processes for bacterial community assembly (Figure 7). The median betaNTI of the soil bacterial community was below -2 in both rice and wheat harvesting periods, indicating that deterministic processes dominated the soil bacterial community. However, the lower betaNTI of the rice harvesting period indicated that deterministic processes contributed more to the formation of soil bacterial community (Figure 7A). The results provided further evidence that environmental factors, especially pH and TOC, significantly influenced the composition of microbial communities during straw decomposition.

Homogeneous selection was the most important process, representing 99.74% of soil bacterial variation at the rice harvest stage. The dominance of environmental filtration is evidenced by the prevalence of anaerobic conditions, mirroring findings from previous research (Li L. et al., 2024).

At the wheat harvest stage, although homogeneous selection was the dominant ecological process (78.31%), heterogeneous selection (6.35%), homogenizing dispersal (10.05%), and ecological drift (5.29%) also contributed to the community (Figure 7B). As soil nutrient content increased following straw incorporation, deterministic processes played a dominant role in shaping the wheat rhizosphere, while random processes also exerted some influence.

## 4 Discussion

China is endowed with abundant straw resources. Straw return has been shown to enhance soil carbon sequestration. As Huang et al. (2024) pointed out, in the long run, this practice can significantly reduce the net global warming potential (NGWP). Straw return supplies supplementary sources of readily available carbon and nitrogen for the soil microbes in the relevant environment. This addition has an impact on the activity and diversity of soil microorganisms (Huang et al., 2022). To promote green agriculture, straw incorporation has become increasingly common in China, especially in northeast China (Xia et al., 2020; Zhu Y. et al., 2023; Coleine et al., 2024). Previous studies have shown that straw return significantly affects the physical and chemical properties of the soil and microbial communities (Bu et al., 2020; Wu et al., 2020; Yan et al., 2020). While some studies have shown that straw return has a significant impact on the soil microbial community, there is little knowledge about soil microbial diversity among different crops and the dynamic changes of soil microbial composition under varying straw return durations. In this study, we evaluated the effects of different durations of straw return on the soil microbial community in the rice-wheat harvesting period and explored the co-occurrence network and assembly mechanism of soil bacterial communities.

Previous research has yielded various results regarding the impact of straw return on the diversity of soil bacteria and fungi in rice or wheat fields. Despite numerous studies investigating straw return on soil microbial diversity and richness, the findings remain inconclusive. While some studies have reported positive influences of straw return on soil microbial communities, enhancing soil bacterial diversity (Luo et al., 2020), others have identified no significant effect or even a decrease in soil microbial diversity (Jin et al., 2020). Wheat straw return substantially increases the diversity of soil bacterial and fungal communities in systems such as wheat-soybean rotations (Yang H. et al., 2022). Additionally, the timing and quantity of straw return impact soil organic matter content and microbial structure. Research has shown that in field experiments involving the return of corn straw, a 33% straw return over 8 years enhances the accumulation of readily available carbon components, promotes microbial growth, and facilitates fungal-mediated soil carbon sequestration (Zhu X. et al., 2023). In this study, we established seven groups according to different straw return durations and found that straw return influenced the alpha diversity of the bacterial community. However, with changes in straw return duration, there was no obvious change in soil alpha diversity in the rice harvesting period; in the wheat harvesting period, the soil alpha diversity increased first, remained stable, and decreased (Figure 2). This indicated that the duration of straw return had a significant effect on soil microbial diversity. Specifically, the duration of straw return significantly impacts the relative abundance of some bacterial taxa (Acidobacteriota, MBNT15, Bacteroidota, Nitrosoirae, and Verrucomicrobiota) (Figure 4). Studies have demonstrated that the main function of Acidobacteria is to degrade plant residues, and their abundance increases in low organic carbon content soils (Liu et al., 2016). This suggests that adding a small amount of straw will increase the acid and low organic carbon content in paddy soil, leading to increased Acidobacteria. Furthermore, one study reported organic carbon content in the paddy soil increased with the amount of straw returned, whereas the abundance of Acidobacteria and Nitrospira decreased (Shahbaz et al., 2017). These studies highlight the complex and variable influence of different types and durations of straw return on soil bacterial and fungal diversity in rice or wheat fields. In addition, existing research (Liu et al., 2022) have demonstrated that long-term straw return leads to an increase in the abundance of microorganisms. However, this effect is seasonal, with the influence of straw return on soil microorganisms being more pronounced in spring compared to autumn. During the growing season, the overall abundance of soil bacteria, archaea, and fungi tends to rise, though not always in a statistically significant manner. Therefore, when conducting future research, it is crucial to consider seasonal factors. A more in-depth discussion on the impacts of straw return microbial community should be carried out.

We found a significant alteration in the microbial community throughout the 3 yeasr period of straw incorporation, potentially due to the level of straw decay. The composting process of straw induces changes in environmental conditions, including high temperature, C/N ratio, and moisture levels (Wei et al., 2018). These changes alter the composition and structure of microorganism communities, resulting in a distinct and homogeneous bacterial community compared to the primary community in the raw material.

Network analysis offers a method to investigate microbial communities by revealing non-random covariant patterns in community organizations (Xu et al., 2023). Analyzing the structural features of the ecological network may identify complex relationships between species and the stability of the ecological network structure (Matchado et al., 2021; Peng Y. et al., 2024). The complexity of the network is demonstrated by the number and density of the nodes and edges (Guseva et al., 2022). In this study, relative to wheat, the rice network analysis showed that the soil bacterial community network was more complex, with increased nodes and variable numbers, average path length, diameter, and modularity index (Figure 6). Given that association in co-occurrence networks implies ecological interactions or niche sharing between microorganisms (Zhai et al., 2024), the primary factors driving the stability and complexity of microbial collinear networks should be further examined in future studies.

Liu et al. (2022) study showed that long-term straw return had more obvious effects on rare species. The comparison of co-occurrence network maps of rare species and dominant species showed that the network constructed with dominant species had fewer connections and less complexity than the network constructed with rare species. Members of rare groups have stronger and finer interconnections. This may be due to the fact that rare taxa take longer to develop synergistic activity, harvesting energy and carbon from more complex organic substrates. It is likely that rare bacterial communities represent more oligotrophic and synergistic bacteria, which form a stronger symbiotic and competitive network with each other. In future studies, the analysis of intercommunity structure of rare species will help to better understand the interaction between microorganisms and straw return.

The living strategies of soil microorganisms, which are propelled by community assembly processes (two main ecological processes, determinism and stochasticity), serve as the fundamental basis for the structuring mechanisms of these microorganisms (Anthony et al., 2020). Moreover, an increasing number of studies are being conducted on these strategies in an attempt to predict the biogeochemical functions of the soil (Liu W. et al., 2020; Zhu et al., 2024; Luan et al., 2020). Determinism, grounded in niche theory, places emphasis on the impacts of environmental filtering and biotic interactions on the abundance and fitness of microorganisms. On the other hand, stochasticity, which is founded on neutral theory, accentuates the influences of unpredictable disturbances, random occurrences of birth and death, as well as dispersal processes on the composition of microbial communities (Ning et al., 2019; Zhu et al., 2024).

Studies on the mechanism of bacterial and fungal community development in rice or wheat soil following straw return have revealed notable findings. The mechanism of bacterial and fungal community construction in rice or wheat soil is influenced by both deterministic and random processes (Liu et al., 2023). Our results demonstrated that the median betaNTI of the soil bacterial community was lower than -2 in both rice and wheat harvesting periods, indicating that deterministic processes dominated the shaping of the soil bacterial community (Figure 7).

The results of Pearson correlation analysis and RDA analysis indicated that environmental factors significantly influenced the diversity and composition of soil bacterial communities, with pH, available potassium, and ammonia nitrogen significantly correlated with bacterial communities at rice harvest stage, whereas total nitrogen, available phosphorus, and nitrate were important at the wheat harvest stage. Nitrogen, ammonia nitrogen, and TOC were significantly associated with bacterial communities. Previous studies have shown that nutrients are critical environmental factors determining the diversity and composition of bacterial communities. Notably, nutrients govern the structure and distribution of bacterial communities, while deterministic processes dominate community assembly (Hao et al., 2024).

Straw return enhances the significance of deterministic selection, which is likely associated with the quantity of SOC. This is because alterations in the organic carbon content can elevate the probability of either homogeneous or variable selection. As a result, the organic carbon content initiates coordinated changes in the assembly of the microbial community (Dini-Andreote et al., 2015). Additionally, Liu et al. (2023) research demonstrated that straw return remarkably enhanced the homogeneous selection within the soil microbial communities in rice-wheat cropping systems. Straw return was found to be accompanied by a simultaneous increase in SOC levels and the influence of deterministic processes. The contribution of homogeneous selection was more significant when both rice straw and wheat straw were returned, compared to the scenarios where only rice straw or only wheat straw was incorporated. Despite a preliminary comprehension of the relationship between microbial communities and SOC, the extent to which the downstream biochemical metabolic activities of microorganisms, influenced by straw return practices, contribute to SOC still needs further clarification for future research.

Certainly, although the deterministic processes dominate at larger scales, stochastic processes also play a significant role in driving alterations within soil microbial communities, especially in wheat systems. The regulation and management of soil microbial communities to enhance soil fertility and crop system yields should consider both deterministic and stochastic factors influencing microbes.

Summarily, this study illustrated the impacts of straw returning on the composition of soil microorganisms and the mechanisms underlying community formation during the harvesting periods of wheat and rice. It also revealed that SOC is one of the key factors influencing changes in the microbial community. Nevertheless, there remains a dearth of evidence regarding how SOC affects the mechanisms of microbial community construction and the interaction between SOC and the microbial community. In future research, it is essential to take into account a wider range of influencing factors, such as the seasonal effects of straw return and the content of soil organic matter. When necessary, multi-omics approaches, including metagenomic, transcriptomic, and metabolomic studies, can be considered. Through the joint analysis of multiple omics data, it will be possible to clarify the impact of straw return on the microbial community and highlight the significance of such practices for sustainable agriculture.

Furthermore, straw return is a principal and efficient way of straw utilization. However, China’s diverse climates, soils, farming practices, and planting systems call for more comprehensive research and evaluation. Going forward, attention should center on innovative straw incorporation methods, such as ditch burial, deep burial with plastic film mulching, and the combination of straw incorporation and fertilization. Moreover, long-term field trials, especially for new incorporation models, are essential.

## 5 Conclusion

Straw incorporation significantly influences soil microbial composition, especially in the wheat harvesting period. Compared with the wheat harvest period, the alpha diversity of the soil bacterial community in the rice harvest period fluctuated steadily in different straw return duration samples and exhibited a trend of first increasing and then decreasing. The difference in soil microbial community composition between wheat and rice was significantly impacted by environmental factors, especially pH and TOC. The analysis of co-occurrence networks uncovered that the network structure of the rice soil microbial community exhibited increased complexity and stability in the wheat group, which is closely associated with the accumulation of TOC in rice cultivation. Additionally, soil microbial communities in wheat and rice are dominated by deterministic processes. This study has described the changes in soil microbial communities in rice and wheat at seven different straw return durations, emphasizing that straw return and how soil microbial communities are shaped by straw return duration inform future strategies for sustainable agriculture and soil fertility recovery.

## Data Availability

The datasets presented in this study can be found in online repositories. The names of the repository/repositories and accession number(s) can be found here: https://www.ncbi.nlm.nih.gov/sra, SRP546496.

## Footnotes

1
http://www.usadellab.org/cms/?page=trimmomatic

2
https://github.com/topics/uclust

3
http://www.arb-silva.de

## References

[B1] Anthony M. A. Crowther T. W. Maynard D. S. van den Hoogen J. Averill C. (2020). Distinct assembly processes and microbial communities constrain soil organic carbon formation. *One Earth* 2 349–360. 10.1016/j.oneear.2020.03.006

[B2] Bao S. D. (2000). *Soil Agro-Chemistrical Analysis*, 3rd Edn. Beijing: China Agriculture Press.

[B3] Bu R. Ren T. Lei M. Liu B. Li X. Cong R. (2020). Tillage and straw-returning practices effect on soil dissolved organic matter, aggregate fraction and bacteria community under rice-rice-rapeseed rotation system. *Agric. Ecosyst. Environ.* 287:106681. 10.1016/j.agee.2019.106681

[B4] Callahan B. J. McMurdie P. J. Rosen M. J. Han A. W. Johnson A. J. A. Holmes S. P. (2016). DADA2: High-resolution sample inference from Illumina amplicon data. *Nat. Methods* 13 581–583. 10.1038/nmeth.3869 27214047 PMC4927377

[B5] Chen C. Wang M. Zhu J. Tang Y. Zhang H. Zhao Q. (2022). Long-term effect of epigenetic modification in plant–microbe interactions: Modification of DNA methylation induced by plant growth-promoting bacteria mediates promotion process. *Microbiome* 10:36. 10.1186/s40168-022-01236-9 35209943 PMC8876431

[B6] Chen L. Sun S. Yao B. Peng Y. Gao C. Qin T. (2022). Effects of straw return and straw biochar on soil properties and crop growth: A review. *Front. Plant Sci.* 13:986763. 10.3389/fpls.2022.986763 36237511 PMC9552067

[B7] Coleine C. Delgado-Baquerizo M. DiRuggiero J. Guirado E. Harfouche A. L. Perez-Fernandez C. (2024). Dryland microbiomes reveal community adaptations to desertification and climate change. *ISME J.* 18:wrae056. 10.1093/ismejo/wrae056 38552152 PMC11031246

[B8] Davison J. Gerz M. Hiiesalu I. Moora M. Semchenko M. Zobel M. (2024). Niche types and community assembly. *Ecol. Lett.* 27:e14327. 10.1111/ele.14327 37819920

[B9] Dini-Andreote F. Stegen J.-C. van Elsas J.-D. Salles J.-F. (2015). Disentangling mechanisms that mediate the balance between stochastic and deterministic processes in microbial succession. *Proc. Natl. Acad. Sci.* 112 E1326–E1332. 10.1073/pnas.1414261112 25733885 PMC4371938

[B10] Edgar R. C. (2010). Search and clustering orders of magnitude faster than BLAST. *Bioinformatics* 26 2460–2461. 10.1093/bioinformatics/btq461 20709691

[B11] Gong Z. T. Chen Z. C. (2007). *Pedogenesis and Soil Taxonomy.* Beijing: Science Press.

[B12] Guseva K. Darcy S. Simon E. Alteio L. V. Montesinos-Navarro A. Kaiser C. (2022). From diversity to complexity: Microbial networks in soils. *Soil Biol. Biochem.* 169:108604. 10.1016/j.soilbio.2022.108604 35712047 PMC9125165

[B13] Hao H. Yue Y. Chen Q. Yang Y. Kuai B. Wang Q. (2024). Effects of an efficient straw decomposition system mediated by Stropharia rugosoannulata on soil properties and microbial communities in forestland. *Sci. Total Environ.* 916:170226. 10.1016/j.scitotenv.2024.170226 38280599

[B14] Huang Q. Zhang G. Song K. Zhu X. Shen W. Xu H. (2022). Dynamic interactions of nitrogen fertilizer and straw application on greenhouse gas emissions and sequestration of soil carbon and nitrogen: A 13-year field study. *Agric. Ecosyst. Environ.* 325:107753.

[B15] Huang T.-T. Wen S.-Y. Zhang M.-X. Pan Y.-P. Chen X.-P. Pu X. (2024). Effect on greenhouse gas emissions (CH4 and N2O) of straw mulching or its incorporation in farmland ecosystems in China. *Sustainable Prod. Consumpt.* 46 223–232. 10.1016/j.spc.2024.02.024

[B16] Huddell A. M. Galford G. L. Tully K. L. Crowley C. Palm C. A. Neill C. (2020). Meta-analysis on the potential for increasing nitrogen losses from intensifying tropical agriculture. *Glob. Change Biol.* 26 1668–1680. 10.1111/gcb.14951 31984585

[B17] Jiang Y. Sun B. Li H. Liu M. Chen L. Zhou S. (2015). Aggregate-related changes in network patterns of nematodes and ammonia oxidizers in an acidic soil. *Soil Biol. Biochem.* 88 101–109. 10.1016/j.soilbio.2015.05.013

[B18] Jin S. Jin W. Dong C. Bai Y. Jin D. Hu Z. (2020). Effects of rice straw and rice straw ash on rice growth and α-diversity of bacterial community in rare-earth mining soils. *Sci. Rep.* 10:10331. 10.1038/s41598-020-67160-w 32587300 PMC7316728

[B19] Kong W. Wei X. Wu Y. Shao M. Zhang Q. Sadowsky M. J. (2022). Afforestation can lower microbial diversity and functionality in deep soil layers in a semiarid region. *Glob. Change Biol.* 28 6086–6101. 10.1111/gcb.16334 35808859

[B20] Li L. Kuzyakov Y. Xu Q. Guo H. Zhu C. Guo J. (2024). Bacterial communities in cropland soils: Taxonomy and functions. *Plant Soil* 497 297–315. 10.1007/s11104-023-06396-7

[B21] Li Y.-Z. Bao X.-L. Zhu X.-F. Deng F.-B. Yang Y.-L. Zhao Y. (2024). Parent material influences soil properties to shape bacterial community assembly processes, diversity, and enzyme-related functions. *Sci. Total Environ.* 927:172064. 10.1016/j.scitotenv.2024.172064 38569968

[B22] Liu B. Arlotti D. Huyghebaert B. Tebbe C.-C. (2022). Disentangling the impact of contrasting agricultural management practices on soil microbial communities-Importance of rare bacterial community members. *Soil Biol. Biochem.* 166:108573. 10.1016/j.soilbio.2022.108573

[B23] Liu J. Fang L. Qiu T. Chen J. Wang H. Liu M. (2023). Crop residue return achieves environmental mitigation and enhances grain yield: A global meta-analysis. *Agron. Sustainable Dev.* 43:78. 10.1007/s13593-023-00928-2

[B24] Liu J. Sui Y. Yu Z. Yao Q. Shi Y. Chu H. (2016). Diversity and distribution patterns of acidobacterial communities in the black soil zone of northeast China. *Soil Biol. Biochem.* 95 212–222. 10.1016/j.soilbio.2015.12.021

[B25] Liu J. Wang Y. Li Y. Liu X. Jiang Y. Fu Y. (2020). Ecosystem N:P stoichiometric ratios determine the catchment surface water N:P ratio through subsurface hydrological processes. *CATENA* 194:104740. 10.1016/j.catena.2020.104740

[B26] Liu W. Graham E. B. Zhong L. Zhang J. Li W. Li Z. (2020). Dynamic microbial assembly processes correspond to soil fertility in sustainable paddy agroecosystems. *Funct. Ecol.* 34 1244–1256. 10.1111/1365-2435.13550

[B27] Liu X. Liu H. Zhang Y. Chen G. Li Z. Zhang M. (2023). Straw return drives soil microbial community assemblage to change metabolic processes for soil quality amendment in a rice-wheat rotation system. *Soil Biol. Biochem.* 185:109131. 10.1016/j.soilbio.2023.109131

[B28] Luan L. Liang C. Chen L. Wang H. Xu Q. Jiang Y. (2020). Coupling bacterial community assembly to microbial metabolism across soil profiles. *mSystems* 5:e00298-20. 10.1128/msystems.00298-20 32518195 PMC7289589

[B29] Luo Y. Iqbal A. He L. Zhao Q. Wei S. Ali I. (2020). Long-term no-tillage and straw retention management enhances soil bacterial community diversity and soil properties in Southern China. *Agronomy* 10:1233.

[B30] Matchado M. S. Lauber M. Reitmeier S. Kacprowski T. Baumbach J. Haller D. (2021). Network analysis methods for studying microbial communities: A mini review. *Comput. Struct. Biotechnol. J.* 19 2687–2698. 10.1016/j.csbj.2021.05.001 34093985 PMC8131268

[B31] Ning D. Deng Y. Tiedje J. M. Zhou J. (2019). A general framework for quantitatively assessing ecological stochasticity. *PNAS* 116 16892–16898. 10.1073/pnas.1904623116 31391302 PMC6708315

[B32] Peng Y. Xu H. Shi J. Wang Z. Lv J. Li L. (2024). Soil microbial composition, diversity, and network stability in intercropping versus monoculture responded differently to drought. *Agric. Ecosyst. Environ.* 365:108915. 10.1016/j.agee.2024.108915

[B33] Peng Z. Qian X. Liu Y. Li X. Gao H. An Y. (2024). Land conversion to agriculture induces taxonomic homogenization of soil microbial communities globally. *Nat. Commun.* 15:3624. 10.1038/s41467-024-47348-8 38684659 PMC11058813

[B34] Qiu L. Zhang Q. Zhu H. Reich P. B. Banerjee S. van der Heijden M. G. A. (2021). Erosion reduces soil microbial diversity, network complexity and multifunctionality. *ISME J.* 15 2474–2489. 10.1038/s41396-021-00913-1 33712698 PMC8319411

[B35] Quast C. Pruesse E. Yilmaz P. Gerken J. Schweer T. Yarza P. (2012). The SILVA ribosomal RNA gene database project: Improved data processing and web-based tools. *Nucleic Acids Res*. 41 D590–D596. 10.1093/nar/gks1219 23193283 PMC3531112

[B36] Shahbaz M. Kuzyakov Y. Sanaullah M. Heitkamp F. Zelenev V. Kumar A. (2017). Microbial decomposition of soil organic matter is mediated by quality and quantity of crop residues: Mechanisms and thresholds. *Biol. Fertility Soils* 53 287–301. 10.1007/s00374-016-1174-9

[B37] Shi Y. Dang K. Dong Y. Feng M. Wang B. Li J. (2020). Soil fungal community assembly processes under long-term fertilization. *Eur. J. Soil Sci.* 71 716–726. 10.1111/ejss.12902

[B38] Siska C. Kechris K. (2017). Differential correlation for sequencing data. *BMC Res. Notes* 10:54. 10.1186/s13104-016-2331-9 28103954 PMC5244536

[B39] Stegen J. C. Lin X. Fredrickson J. K. Chen X. Kennedy D. W. Murray C. J. (2013). Quantifying community assembly processes and identifying features that impose them. *ISME J.* 7 2069–2079. 10.1038/ismej.2013.93 23739053 PMC3806266

[B40] Stegen J. C. Lin X. Konopka A. E. Fredrickson J. K. (2012). Stochastic and deterministic assembly processes in subsurface microbial communities. *ISME J.* 6 1653–1664. 10.1038/ismej.2012.22 22456445 PMC3498916

[B41] Sun D. Bian G. Zhang K. Liu N. Yin Y. Hou Y. (2024). Early-life ruminal microbiome-derived indole-3-carboxaldehyde and prostaglandin D2 are effective promoters of rumen development. *Genome Biol.* 25:64. 10.1186/s13059-024-03205-x 38438919 PMC10910749

[B42] Sun W. Xiao E. Pu Z. Krumins V. Dong Y. Li B. (2018). Paddy soil microbial communities driven by environment- and microbe-microbe interactions: A case study of elevation-resolved microbial communities in a rice terrace. *Sci. Total Environ.* 612 884–893. 10.1016/j.scitotenv.2017.08.275 28886540

[B43] Wang E. Lin X. Tian L. Wang X. Ji L. Jin F. (2021). Effects of short-term rice straw return on the soil microbial community. *Agriculture* 11:561.

[B44] Wang Y. Yu Q. Zheng C. Wang Y. Chen H. Dong S. (2024). The impact of microbial inoculants on large-scale composting of straw and manure under natural low-temperature conditions. *Bioresour. Technol.* 400:130696. 10.1016/j.biortech.2024.130696 38614144

[B45] Wei H. Wang L. Hassan M. Xie B. (2018). Succession of the functional microbial communities and the metabolic functions in maize straw composting process. *Bioresour. Technol.* 256 333–341. 10.1016/j.biortech.2018.02.050 29459320

[B46] Wei L. Ge T. Zhu Z. Ye R. Peñuelas J. Li Y. (2022). Paddy soils have a much higher microbial biomass content than upland soils: A review of the origin, mechanisms, and drivers. *Agric. Ecosyst. Environ.* 326:107798. 10.1016/j.agee.2021.107798

[B47] Wu L. Ma H. Zhao Q. Zhang S. Wei W. Ding X. (2020). Changes in soil bacterial community and enzyme activity under five years straw returning in paddy soil. *Eur. J. Soil Biol.* 100:103215. 10.1016/j.ejsobi.2020.103215

[B48] Xia Y. Chen X. Zheng X. Deng S. Hu Y. Zheng S. (2020). Preferential uptake of hydrophilic and hydrophobic compounds by bacteria and fungi in upland and paddy soils. *Soil Biol. Biochem.* 148:107879. 10.1016/j.soilbio.2020.107879

[B49] Xiong W. Song Y. Yang K. Gu Y. Wei Z. Kowalchuk G. A. (2020). Rhizosphere protists are key determinants of plant health. *Microbiome* 8:27. 10.1186/s40168-020-00799-9 32127034 PMC7055055

[B50] Xu P. Stirling E. Xie H. Li W. Lv X. Matsumoto H. (2023). Continental scale deciphering of microbiome networks untangles the phyllosphere homeostasis in tea plant. *J. Adv. Res.* 44 13–22. 10.1016/j.jare.2022.04.002 36725184 PMC9936419

[B51] Xu Q. Zhang H. Vandenkoornhuyse P. Guo S. Kuzyakov Y. Shen Q. (2024). Carbon starvation raises capacities in bacterial antibiotic resistance and viral auxiliary carbon metabolism in soils. *Proc. Natl. Acad. Sci.* 121:e2318160121. 10.1073/pnas.2318160121 38598339 PMC11032446

[B52] Yan S. Song J. Fan J. Yan C. Dong S. Ma C. (2020). Changes in soil organic carbon fractions and microbial community under rice straw return in Northeast China. *Glob. Ecol. Conserv.* 22:e00962. 10.1016/j.gecco.2020.e00962

[B53] Yang H. Fang C. Meng Y. Dai Y. Liu J. (2021). Long-term ditch-buried straw return increases functionality of soil microbial communities. *CATENA* 202:105316. 10.1016/j.catena.2021.105316

[B54] Yang H. Zhao Y. Ma J. Rong Z. Chen J. Wang Y. (2022). Wheat straw return influences soybean root-associated bacterial and fungal microbiota in a wheat–soybean rotation system. *Microorganisms* 10:667. 35336243 10.3390/microorganisms10030667 PMC8951542

[B55] Yang L. Muhammad I. Chi Y. X. Wang D. Zhou X. B. (2022). Straw return and nitrogen fertilization to maize regulate soil properties, microbial community, and enzyme activities under a dual cropping system. *Front. Microbiol.* 13:823963. 10.3389/fmicb.2022.823963 35369510 PMC8965350

[B56] Yuan L. Gao Y. Mei Y. Liu J. Kalkhajeh Y. K. Hu H. (2023). Effects of continuous straw returning on bacterial community structure and enzyme activities in rape-rice soil aggregates. *Sci. Rep.* 13:2357. 10.1038/s41598-023-28747-1 36759519 PMC9911641

[B57] Zhai C. Han L. Xiong C. Ge A. Yue X. Li Y. (2024). Soil microbial diversity and network complexity drive the ecosystem multifunctionality of temperate grasslands under changing precipitation. *Sci. Total Environ.* 906:167217. 10.1016/j.scitotenv.2023.167217 37751844

[B58] Zhang M. Dang P. Haegeman B. Han X. Wang X. Pu X. (2024). The effects of straw return on soil bacterial diversity and functional profiles: A meta-analysis. *Soil Biol. Biochem.* 195:109484. 10.1016/j.soilbio.2024.109484

[B59] Zhang S. Zhang G. Wang D. Liu Q. (2021). Long-term straw return with N addition alters reactive nitrogen runoff loss and the bacterial community during rice growth stages. *J. Environ. Manag.* 292:112772. 10.1016/j.jenvman.2021.112772 34022644

[B60] Zhang W. Shen Z. Shao Y. Shi L. Liu S. Shi N. (2020). Soil biota and sustainable agriculture: A review. *Acta Ecol. Sin.* 40 3183–3206. 10.5846/stxb201903310622

[B61] Zhou X. Xiao C. Zhang B. Chen T. Yang X. (2024). Effects of microplastics on carbon release and microbial community in mangrove soil systems. *J. Hazardous Mater.* 465:133152. 10.1016/j.jhazmat.2023.133152 38056259

[B62] Zhu X. Min K. Feng K. Xie H. He H. Zhang X. (2024). Microbial necromass contribution to soil carbon storage via community assembly processes. *Sci. Total Environ.* 951:175749. 10.1016/j.scitotenv.2024.175749 39187085

[B63] Zhu X. Xie H. Masters M. D. Rui Y. Luo Y. He H. (2023). Microorganisms, their residues, and soil carbon storage under a continuous maize cropping system with eight years of variable residue retention. *Appl. Soil Ecol.* 187:104846. 10.1016/j.apsoil.2023.104846

[B64] Zhu Y. Wang L. Song X. Li X. Ma J. Chen F. (2023). Changes in abundant and rare microbial taxa that dominated the formation of soil carbon pool during short-term dryland-to-paddy conversion. *Carbon Res.* 2:26. 10.1007/s44246-023-00060-6

